# 3-{[4-(4-Pyrid­yl)pyrimidin-2-yl]sulfanylmeth­yl}benzoic acid

**DOI:** 10.1107/S1600536808036799

**Published:** 2008-11-13

**Authors:** Hai-Bin Zhu, Hai Wang, Jun-Feng Ji

**Affiliations:** a School of Chemistry and Chemical Engineering, Southeast University, Nanjing, People’s Republic of China

## Abstract

The title compound, C_17_H_13_N_3_O_2_S, was prepared by reaction of 4-(4-pyrid­yl)pyrimidine-2-thiol with 3-(bromo­meth­yl)benzoic acid under basic conditions. Each pair of mol­ecules is mutually linked *via* O—H⋯N hydrogen bonds, forming a dimer. The packing of the dimers is stablized by C—H⋯π inter­actions involving the methyl­ene unit of the –CH_2_S– linkage and benzene rings.

## Related literature

For monodentate and chelating ligands, see: Raper (1996 ▶). For the structures of binuclear and polynuclear complexes with bridging heterocyclic thio­nate ligands, see: Raper (1997 ▶). For O—H⋯N inter­actions, see: Han *et al.* (2008 ▶). For C—H⋯π inter­actions, see: Choi *et al.* (2008 ▶).
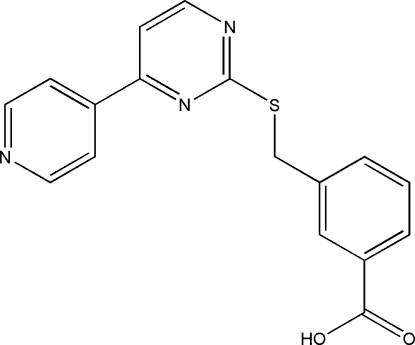


## Experimental

### 

#### Crystal data

C_17_H_13_N_3_O_2_S
*M* *_r_* = 323.36
Triclinic, 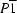

*a* = 4.4130 (9) Å
*b* = 10.458 (2) Å
*c* = 16.432 (3) Å
α = 87.79 (3)°
β = 89.90 (3)°
γ = 80.48 (3)°
*V* = 747.4 (3) Å^3^
*Z* = 2
Mo *K*α radiation
μ = 0.23 mm^−1^
*T* = 298 (2) K
0.30 × 0.10 × 0.10 mm

#### Data collection

Enraf–Nonius CAD-4 diffractometer
Absorption correction: ψ scan (North *et al.*, 1968 ▶) *T*_min_ = 0.934, *T*_max_ = 0.977
3108 measured reflections
2724 independent reflections
1685 reflections with *I* > 2σ(*I*)
*R*_int_ = 0.048
3 standard reflections every 200 reflections intensity decay: 1%

#### Refinement

*R*[*F* ^2^ > 2σ(*F* ^2^)] = 0.071
*wR*(*F* ^2^) = 0.170
*S* = 1.04
2724 reflections
208 parameters
H-atom parameters constrained
Δρ_max_ = 0.35 e Å^−3^
Δρ_min_ = −0.25 e Å^−3^

### 

Data collection: *CAD-4 Software* (Enraf–Nonius, 1989 ▶); cell refinement: *CAD-4 Software*; data reduction: *XCAD4* (Harms & Wocadlo, 1995 ▶); program(s) used to solve structure: *SHELXS97* (Sheldrick, 2008 ▶); program(s) used to refine structure: *SHELXL97* (Sheldrick, 2008 ▶); molecular graphics: *SHELXTL* (Sheldrick, 2008 ▶) and *DIAMOND* (Brandenburg, 1999 ▶); software used to prepare material for publication: *SHELXTL* and *PLATON* (Spek, 2003 ▶).

## Supplementary Material

Crystal structure: contains datablocks I, global. DOI: 10.1107/S1600536808036799/si2127sup1.cif

Structure factors: contains datablocks I. DOI: 10.1107/S1600536808036799/si2127Isup2.hkl

Additional supplementary materials:  crystallographic information; 3D view; checkCIF report

## Figures and Tables

**Table 1:** Hydrogen-bond geometry (Å, °)

*D*—H⋯*A*	*D*—H	H⋯*A*	*D*⋯*A*	*D*—H⋯*A*
O2—H2*A*⋯N1^i^	0.82	1.84	2.659 (5)	174
C10—H10*B*⋯*Cg*3^ii^	0.97	2.56	3.398 (5)	145

Symmetry codes: (i) 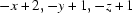; (ii) 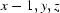. *Cg*3 is the centroid of the phenyl ring.

**Fig. 1.**
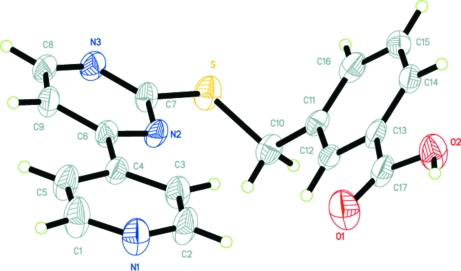
The molecular structure of the title compound with 30% displacement ellipsoids.

**Fig. 2.**
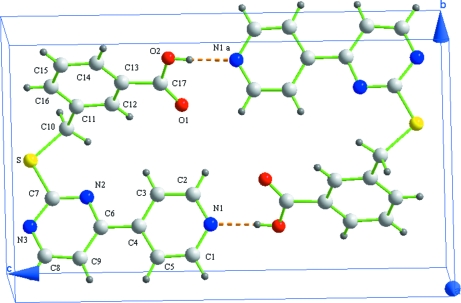
The hydrogen bonded dimer with O—H···N interactions (dotted lines) [symmetry code: -x + 2, -y + 1, -z + 1].

**Fig. 3.**
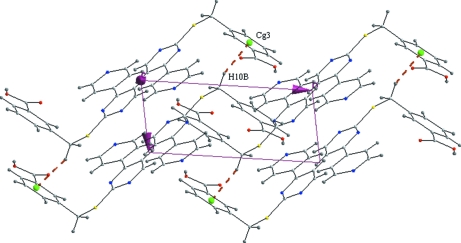
A section of the hydrogen bonded dimers viewed down the c axis. C—H···π interactions are shown as dotted lines. Cg3 denotes the phenyl ring centroid [symmetry code: x - 1, y, z].

## supplementary crystallographic information

### Comment

Heterocyclic thionates (Raper, 1996, 1997) have been extensively studied due to not only their versatile coordination modes but also their close biological relativity. Herein, we report the crystal structure of a new heterocyclic thionate derivative, 3-((4-(4-pyridyl)pyrimidin-2-ylthio)methyl)benzoic acid.

In the molecular structure of the title compound (Fig.1), the pyrimidinyl and pyridinyl rings are not coplanar with the dihedral angle of 11.17 (3)*o*. Each pair of compound molecules is linked each other *via* O—H···N hydrogen bonds (Han *et.al*., 2008) to form a dimer (Fig. 2 and Table 1), and the packing of the dimers is stablized by C—H···π interactions (Choi *et al.*, 2008) between a methylene H atom of the –CH_2_S– linkage and the phenyl ring, with a C10—H10B···*Cg*3^ii ^separation of 2.56 Å (Fig. 3 and Table 1; *Cg*3 is the centroid of the C11/C12/C13/C14/C15/C16 phenyl ring, symmetry code as in Fig. 3).

### Experimental

The mixture of 4-(4-pyridyl)pyrimidine-2-thiol (0.1890 g,1.0 mmol), 3-(bromomethyl)benzoic acid (0.2150 g,1.0 mmol), NaOH (0.0400 g,1.0 mmol) in 30 ml of H_2_O was refluxed for 10 h. After cooled to room temperature, the product was filtered and dried in vaccum. The single crystals suitable for X-ray diffraction were obtained by slow evaporation of the title compound in DMF solution.

### Refinement

All H atoms were positioned geometrically and allowed to ride on their parent atoms, with C—H = 0.93 Å for aromatic H atoms, 0.97 Å for methylene H atoms and O—H = 0.82 Å, respectively, and with with *U*iso(H) = 1.2*U*eq (C) for aromatic and methylene, *U*iso(H) =1.5*U*eq (O).

### Crystal data

**Table:**

C_17_H_13_N_3_O_2_S	*Z* = 2
*M**_r_* = 323.36	*F*_000_ = 336
Triclinic, *P*1	*D*_x_ = 1.437 Mg m^−^^3^
Hall symbol: -P 1	Mo *K*α radiation λ = 0.71073 Å
*a* = 4.4130 (9) Å	Cell parameters from 25 reflections
*b* = 10.458 (2) Å	θ = 9–12º
*c* = 16.432 (3) Å	µ = 0.23 mm^−^^1^
α = 87.79 (3)º	*T* = 298 (2) K
β = 89.90 (3)º	Prism, colorless
γ = 80.48 (3)º	0.30 × 0.10 × 0.10 mm
*V* = 747.4 (3) Å^3^	

### Data collection

**Table:**

Enraf–Nonius CAD-4 diffractometer	*R*_int_ = 0.048
Radiation source: fine-focus sealed tube	θ_max_ = 25.3º
Monochromator: graphite	θ_min_ = 1.2º
*T* = 298(2) K	*h* = 0→5
ω/2θ scans	*k* = −12→12
Absorption correction: ψ scan(North *et al.*, 1968)	*l* = −19→19
*T*_min_ = 0.934, *T*_max_ = 0.977	3 standard reflections
3108 measured reflections	every 200 reflections
2724 independent reflections	intensity decay: 1%
1685 reflections with *I* > 2σ(*I*)	

### Refinement

**Table:**

Refinement on *F*^2^	Secondary atom site location: difference Fourier map
Least-squares matrix: full	Hydrogen site location: inferred from neighbouring sites
*R*[*F*^2^ > 2σ(*F*^2^)] = 0.071	H-atom parameters constrained
*wR*(*F*^2^) = 0.170	*w* = 1/[σ^2^(*F*_o_^2^) + (0.05*P*)^2^ + *P*] where *P* = (*F*_o_^2^ + 2*F*_c_^2^)/3
*S* = 1.04	(Δ/σ)_max_ < 0.001
2724 reflections	Δρ_max_ = 0.35 e Å^−^^3^
208 parameters	Δρ_min_ = −0.25 e Å^−^^3^
Primary atom site location: structure-invariant direct methods	Extinction correction: none

### Special details

**Table:**

Geometry. All e.s.d.'s (except the e.s.d. in the dihedral angle between two l.s. planes) are estimated using the full covariance matrix. The cell e.s.d.'s are taken into account individually in the estimation of e.s.d.'s in distances, angles and torsion angles; correlations between e.s.d.'s in cell parameters are only used when they are defined by crystal symmetry. An approximate (isotropic) treatment of cell e.s.d.'s is used for estimating e.s.d.'s involving l.s. planes.
Refinement. Refinement of *F*^2^ against ALL reflections. The weighted *R*-factor *wR* and goodness of fit *S* are based on *F*^2^, conventional *R*-factors *R* are based on *F*, with *F* set to zero for negative *F*^2^. The threshold expression of *F*^2^ > σ(*F*^2^) is used only for calculating *R*-factors(gt) *etc*. and is not relevant to the choice of reflections for refinement. *R*-factors based on *F*^2^ are statistically about twice as large as those based on *F*, and *R*- factors based on ALL data will be even larger.

### Fractional atomic coordinates and isotropic or equivalent isotropic displacement parameters (Å^2^)

**Table:**

	*x*	*y*	*z*	*U*_iso_*/*U*_eq_	
S	0.1838 (3)	0.34695 (12)	0.92472 (7)	0.0633 (4)	
O1	0.6060 (10)	0.6024 (3)	0.5857 (2)	0.0916 (13)	
O2	0.6987 (9)	0.7953 (3)	0.61531 (19)	0.0795 (11)	
H2A	0.7614	0.7948	0.5683	0.119*	
N1	1.0847 (10)	0.1910 (4)	0.5353 (2)	0.0737 (12)	
N2	0.5470 (7)	0.2426 (3)	0.80536 (18)	0.0457 (8)	
N3	0.5608 (9)	0.1325 (4)	0.9366 (2)	0.0597 (10)	
C1	1.1506 (13)	0.0803 (5)	0.5789 (3)	0.0832 (17)	
H1B	1.2665	0.0098	0.5544	0.100*	
C2	0.9037 (14)	0.2874 (5)	0.5702 (3)	0.0819 (17)	
H2B	0.8459	0.3649	0.5403	0.098*	
C3	0.7973 (11)	0.2779 (4)	0.6488 (3)	0.0635 (13)	
H3B	0.6757	0.3490	0.6713	0.076*	
C4	0.8710 (10)	0.1634 (4)	0.6942 (2)	0.0506 (10)	
C5	1.0591 (12)	0.0636 (5)	0.6570 (3)	0.0693 (14)	
H5A	1.1230	−0.0148	0.6854	0.083*	
C6	0.7669 (10)	0.1494 (4)	0.7790 (2)	0.0470 (10)	
C7	0.4682 (9)	0.2307 (4)	0.8824 (2)	0.0462 (10)	
C8	0.7742 (11)	0.0419 (4)	0.9077 (2)	0.0577 (12)	
H8A	0.8544	−0.0276	0.9425	0.069*	
C9	0.8839 (10)	0.0436 (4)	0.8303 (3)	0.0558 (11)	
H9A	1.0315	−0.0232	0.8123	0.067*	
C10	0.0460 (9)	0.4535 (4)	0.8386 (3)	0.0566 (11)	
H10A	0.0430	0.4012	0.7912	0.068*	
H10B	−0.1644	0.4929	0.8493	0.068*	
C11	0.2261 (8)	0.5595 (4)	0.8179 (2)	0.0449 (9)	
C12	0.3212 (9)	0.5779 (4)	0.7389 (2)	0.0448 (9)	
H12A	0.2784	0.5227	0.6991	0.054*	
C13	0.4809 (10)	0.6787 (4)	0.7186 (2)	0.0516 (11)	
C14	0.5392 (10)	0.7623 (4)	0.7761 (2)	0.0516 (10)	
H14A	0.6428	0.8305	0.7619	0.062*	
C15	0.4440 (10)	0.7455 (4)	0.8553 (3)	0.0544 (11)	
H15A	0.4859	0.8015	0.8948	0.065*	
C16	0.2852 (10)	0.6446 (4)	0.8758 (3)	0.0575 (12)	
H16A	0.2182	0.6343	0.9290	0.069*	
C17	0.5941 (11)	0.6869 (4)	0.6328 (3)	0.0579 (12)	

### Atomic displacement parameters (Å^2^)

**Table:**

	*U*^11^	*U*^22^	*U*^33^	*U*^12^	*U*^13^	*U*^23^
S	0.0761 (8)	0.0604 (8)	0.0493 (7)	−0.0024 (6)	0.0165 (5)	0.0109 (5)
O1	0.161 (4)	0.067 (2)	0.0460 (19)	−0.016 (2)	0.026 (2)	−0.0079 (18)
O2	0.125 (3)	0.060 (2)	0.054 (2)	−0.019 (2)	0.0138 (19)	0.0092 (16)
N1	0.104 (3)	0.062 (3)	0.051 (2)	0.000 (2)	0.025 (2)	0.003 (2)
N2	0.056 (2)	0.0419 (19)	0.0373 (18)	−0.0057 (16)	0.0016 (15)	0.0116 (14)
N3	0.087 (3)	0.049 (2)	0.040 (2)	−0.006 (2)	−0.0012 (18)	0.0092 (17)
C1	0.119 (5)	0.054 (3)	0.067 (3)	0.015 (3)	0.027 (3)	0.000 (3)
C2	0.136 (5)	0.057 (3)	0.042 (3)	0.012 (3)	0.011 (3)	0.013 (2)
C3	0.091 (4)	0.048 (3)	0.047 (3)	0.002 (2)	0.010 (2)	0.005 (2)
C4	0.063 (3)	0.041 (2)	0.045 (2)	−0.002 (2)	−0.0035 (19)	0.0036 (18)
C5	0.094 (4)	0.054 (3)	0.051 (3)	0.013 (3)	0.017 (2)	0.011 (2)
C6	0.062 (3)	0.039 (2)	0.039 (2)	−0.007 (2)	−0.0010 (18)	0.0058 (17)
C7	0.056 (2)	0.041 (2)	0.041 (2)	−0.0115 (19)	0.0064 (18)	0.0108 (18)
C8	0.085 (3)	0.046 (3)	0.040 (2)	−0.007 (2)	−0.010 (2)	0.013 (2)
C9	0.074 (3)	0.037 (2)	0.052 (3)	0.004 (2)	−0.002 (2)	0.0017 (19)
C10	0.040 (2)	0.060 (3)	0.065 (3)	0.001 (2)	0.007 (2)	0.009 (2)
C11	0.033 (2)	0.046 (2)	0.051 (2)	0.0036 (17)	−0.0046 (17)	0.0045 (19)
C12	0.049 (2)	0.041 (2)	0.040 (2)	0.0058 (18)	−0.0048 (17)	0.0020 (17)
C13	0.064 (3)	0.040 (2)	0.044 (2)	0.011 (2)	−0.0021 (19)	0.0105 (19)
C14	0.065 (3)	0.039 (2)	0.048 (2)	0.000 (2)	0.001 (2)	0.0047 (19)
C15	0.069 (3)	0.043 (2)	0.049 (3)	−0.002 (2)	−0.006 (2)	−0.0028 (19)
C16	0.064 (3)	0.060 (3)	0.040 (2)	0.016 (2)	0.008 (2)	0.002 (2)
C17	0.074 (3)	0.044 (3)	0.050 (3)	0.005 (2)	0.001 (2)	0.010 (2)

### Geometric parameters (Å, °)

**Table:**

S—C7	1.759 (4)	C5—H5A	0.9300
S—C10	1.809 (4)	C6—C9	1.393 (5)
O1—C17	1.191 (5)	C8—C9	1.360 (6)
O2—C17	1.314 (5)	C8—H8A	0.9300
O2—H2A	0.8200	C9—H9A	0.9300
N1—C2	1.327 (6)	C10—C11	1.496 (6)
N1—C1	1.327 (6)	C10—H10A	0.9700
N2—C7	1.318 (5)	C10—H10B	0.9700
N2—C6	1.341 (5)	C11—C12	1.379 (5)
N3—C8	1.323 (5)	C11—C16	1.382 (6)
N3—C7	1.344 (5)	C12—C13	1.392 (6)
C1—C5	1.358 (6)	C12—H12A	0.9300
C1—H1B	0.9300	C13—C14	1.365 (6)
C2—C3	1.380 (6)	C13—C17	1.499 (6)
C2—H2B	0.9300	C14—C15	1.381 (5)
C3—C4	1.378 (5)	C14—H14A	0.9300
C3—H3B	0.9300	C15—C16	1.390 (6)
C4—C5	1.383 (6)	C15—H15A	0.9300
C4—C6	1.476 (5)	C16—H16A	0.9300
			
C7—S—C10	103.54 (19)	C8—C9—H9A	121.3
C17—O2—H2A	109.5	C6—C9—H9A	121.3
C2—N1—C1	116.5 (4)	C11—C10—S	116.1 (3)
C7—N2—C6	115.9 (3)	C11—C10—H10A	108.3
C8—N3—C7	113.1 (3)	S—C10—H10A	108.3
N1—C1—C5	124.1 (4)	C11—C10—H10B	108.3
N1—C1—H1B	118.0	S—C10—H10B	108.3
C5—C1—H1B	118.0	H10A—C10—H10B	107.4
N1—C2—C3	123.0 (4)	C12—C11—C16	118.9 (4)
N1—C2—H2B	118.5	C12—C11—C10	120.0 (4)
C3—C2—H2B	118.5	C16—C11—C10	121.0 (4)
C4—C3—C2	120.1 (4)	C11—C12—C13	120.4 (4)
C4—C3—H3B	119.9	C11—C12—H12A	119.8
C2—C3—H3B	119.9	C13—C12—H12A	119.8
C3—C4—C5	116.3 (4)	C14—C13—C12	120.4 (4)
C3—C4—C6	122.1 (4)	C14—C13—C17	122.4 (4)
C5—C4—C6	121.6 (4)	C12—C13—C17	117.1 (4)
C1—C5—C4	119.9 (4)	C13—C14—C15	119.8 (4)
C1—C5—H5A	120.1	C13—C14—H14A	120.1
C4—C5—H5A	120.1	C15—C14—H14A	120.1
N2—C6—C9	120.2 (4)	C14—C15—C16	119.8 (4)
N2—C6—C4	117.1 (3)	C14—C15—H15A	120.1
C9—C6—C4	122.6 (4)	C16—C15—H15A	120.1
N2—C7—N3	128.7 (4)	C11—C16—C15	120.6 (4)
N2—C7—S	120.5 (3)	C11—C16—H16A	119.7
N3—C7—S	110.6 (3)	C15—C16—H16A	119.7
N3—C8—C9	124.3 (4)	O1—C17—O2	122.4 (4)
N3—C8—H8A	117.9	O1—C17—C13	124.5 (4)
C9—C8—H8A	117.9	O2—C17—C13	113.0 (4)
C8—C9—C6	117.5 (4)		
			
C2—N1—C1—C5	−3.9 (9)	N3—C8—C9—C6	1.3 (7)
C1—N1—C2—C3	2.9 (9)	N2—C6—C9—C8	−2.2 (6)
N1—C2—C3—C4	−1.9 (9)	C4—C6—C9—C8	179.0 (4)
C2—C3—C4—C5	1.6 (7)	C7—S—C10—C11	−84.7 (3)
C2—C3—C4—C6	178.9 (5)	S—C10—C11—C12	129.7 (3)
N1—C1—C5—C4	3.9 (9)	S—C10—C11—C16	−53.9 (5)
C3—C4—C5—C1	−2.5 (8)	C16—C11—C12—C13	1.6 (5)
C6—C4—C5—C1	−179.9 (5)	C10—C11—C12—C13	178.1 (4)
C7—N2—C6—C9	4.1 (6)	C11—C12—C13—C14	−1.5 (6)
C7—N2—C6—C4	−177.0 (4)	C11—C12—C13—C17	176.1 (4)
C3—C4—C6—N2	13.3 (6)	C12—C13—C14—C15	1.1 (6)
C5—C4—C6—N2	−169.5 (4)	C17—C13—C14—C15	−176.3 (4)
C3—C4—C6—C9	−167.9 (4)	C13—C14—C15—C16	−1.0 (6)
C5—C4—C6—C9	9.3 (7)	C12—C11—C16—C15	−1.4 (6)
C6—N2—C7—N3	−5.8 (6)	C10—C11—C16—C15	−177.8 (4)
C6—N2—C7—S	179.7 (3)	C14—C15—C16—C11	1.1 (6)
C8—N3—C7—N2	4.8 (7)	C14—C13—C17—O1	163.6 (5)
C8—N3—C7—S	179.7 (3)	C12—C13—C17—O1	−14.0 (7)
C10—S—C7—N2	4.9 (4)	C14—C13—C17—O2	−12.5 (6)
C10—S—C7—N3	−170.5 (3)	C12—C13—C17—O2	170.0 (4)
C7—N3—C8—C9	−2.2 (7)		

### Hydrogen-bond geometry (Å, °)

**Table:**

*D*—H···*A*	*D*—H	H···*A*	*D*···*A*	*D*—H···*A*
O2—H2A···N1^i^	0.82	1.84	2.659 (5)	174
C10—H10B···Cg3^ii^	0.97	2.56	3.398 (5)	145

Symmetry codes: (i) −*x*+2, −*y*+1, −*z*+1; (ii) *x*−1, *y*, *z*.
